# Video‐Assisted Thoracoscopic Surgery (VATS) Versus Thoracotomy in the Treatment of Pulmonary Hydatid Cysts: A Randomized Controlled Trial

**DOI:** 10.1002/hsr2.70668

**Published:** 2025-06-11

**Authors:** Fahmi H. Kakamad

**Affiliations:** ^1^ College of Medicine Sulaimani University Sulaymaniyah Kurdistan Iraq; ^2^ Smart Health Tower Sulaymaniyah Kurdistan Iraq; ^3^ Kscien Organization for Scientific Research (Middle East office) Sulaymaniyah Kurdistan Iraq

**Keywords:** echinococcosis, lung hydatid disease, minimally invasive surgery, surgical intervention, VATS

## Abstract

**Background and Aims:**

Pulmonary hydatid cysts (PHC), resulting from infection by *Echinococcus granulosus* larvae, represent a substantial health risk in livestock‐raising areas, often causing respiratory symptoms and even death. The primary treatment for PHC involves surgical excision, either through open thoracotomy or video‐assisted thoracoscopic surgery (VATS). However, the superiority of one method over the other remains debatable. The present study aims to compare VATS and thoracotomy outcomes in managing PHC.

**Methods:**

A trial involving 50 patients was conducted to compare VATS and thoracotomy in managing PHCs. Randomization was implemented, and data collection was performed in a blinded manner. Descriptive and regression analyses were utilized to evaluate the data and find potential confounders.

**Results:**

Patient data were almost evenly distributed across groups. The VATS group had lower postoperative pain levels and return time to work in comparison to those who underwent open thoracotomy. It reached a significant difference in correlation analysis. However, other factors such as age, sex, comorbidities, smoking status, and the size and number of cysts exhibited minimal correlations.

**Conclusion:**

This randomized controlled trial indicates that VATS may provide benefits in alleviating postoperative pain and expediting the resumption of work activities. However, larger‐scale studies are necessary to provide comprehensive clinical recommendations.

## 1 Introduction

Pulmonary hydatid cysts (PHC), resulting from infection by the larval stage of *Echinococcus granulosus* [1, 2], pose a substantial health concern in livestock‐raising areas like Central Asia, Africa, the Middle East, and South America [2, 3]. The disease is characterized by a long incubation period, often spanning several years, during which the hydatid cyst (HC) must attain a considerable size before eliciting clinical symptoms [2]. The disease typically presents with cough (60%), dyspnea (31%), hemoptysis (26%), chest pain (14%), and fever with chills (27%) [1, 2, 4]. Accurate diagnosis relies on essential imaging techniques, including chest X‐ray, computed tomography scan (CT), and magnetic resonance imaging [5, 6]. Chest radiography is employed as the preliminary examination to detect PHC, while CT is the principal diagnostic modality [5]. The cysts are commonly observed as singular or multiple well‐defined or oval‐shaped lesions in imaging assessments [2]. Nevertheless, recognizing the presence of specified antibodies like IgG1 and IgG4 can only be detectable in about 50% of patients with PHC [1, 4]. The cornerstone treatment for PHC is surgical removal whenever possible. Several surgical approaches exist, such as wedge resection, lobectomy, capitonnage, pericystectomy, and intact endocystectomy [7]. Open surgery (thoracotomy) and video‐assisted thoracoscopic surgery (VATS) are the two prevailing surgical methods employed in the management of PHC [8, 9]. Open thoracotomy, the conventional technique, entails a substantial chest wall incision, facilitating proper access to the afflicted lung. During this procedure, the surgeon excises the cyst(s) and may undertake supplementary repairs to the lung tissue if necessary [10]. In contrast, VATS is a less invasive method requiring one or a few small incisions [11]. VATS offers several advantages over thoracotomy. It is associated with shorter operative times [12, 13] and significantly reduced perioperative blood loss [12]. While overall complication rates between VATS and thoracotomy are comparable [12], thoracotomy is more likely to cause extensive complications, such as wound infections and long‐term pain, due to larger incisions and greater chest wall trauma. In contrast, VATS results in fewer wound healing complications and lower postoperative pain levels, attributed to its smaller incisions [8, 13]. Additionally, VATS facilitates faster recovery, allowing patients to resume normal activities more quickly, primarily due to reduced tissue trauma and improved pain management [4]. Despite these advantages, the superiority of VATS over thoracotomy remains controversial due to the limited availability of robust evidence, as current knowledge is primarily derived from observational and retrospective studies [8, 12, 13]. This randomized controlled trial aims to compare treatment outcomes between VATS and open thoracotomy in managing PHC, providing greater clarity on this debatable topic.

## 2 Methods

### 2.1 Setting and Study Designs

This study was a randomized controlled trial designed according to the CONSORT guidelines to assess the efficacy of VATS versus thoracotomy in the PHC treatment [14]. Randomization was executed through the utilization of a random number generator, whereby odd numbers were designated for the thoracotomy approach, while even numbers were allocated for VATS. This approach guaranteed the impartial allocation of patients to the groups. Despite the data collection being blinded to the treatment assignments, it was impossible to blind the operating surgeon due to the inherent nature of the surgical techniques. The study spanned 3 years, from March 2020 to March 2023. All the allocated patients in both groups consented to participate and publish their data in the study. The primary outcomes assessed were intraoperative cyst rupture, relapse, and survival, whereas secondary outcomes included postoperative pain score, hospital stay, occurrence of postoperative complications, and return time to work.

#### 2.1.1 Trial Registry and Ethical Approval

In compliance with the Declaration of Helsinki, the trial was registered in the Research Registry, with the registration number Research Registry 8813. The study was approved by the ethics committee of the College of Medicine at the University of Sulaimani, with approval number 23, on August 24, 2023.

#### 2.1.2 Sample Size

The study was a pilot trial, so a small sample size of 50 patients was maintained to evaluate the safety, feasibility, and potential superiority of VATS in managing PHC.

#### 2.1.3 Eligibility Criteria

Any case presenting with a suspected PHC with a size of at least 3 cm was included in the study. Patients who underwent simultaneous operations for other types of HC, such as hepatic HC, and those with PHC smaller than 3 cm, emergency conditions such as tension pneumothorax, and cases of sepsis were excluded from the study.

#### 2.1.4 Preoperative Diagnosis

Suspected HC cases underwent a comprehensive evaluation, including a blood investigation, chest X‐ray, and contrast‐enhanced CT scan. In instances where the diagnosis was uncertain, immunological tests for HC were done to confirm the diagnosis [15].

#### 2.1.5 Operation Details

**Figure 1:**
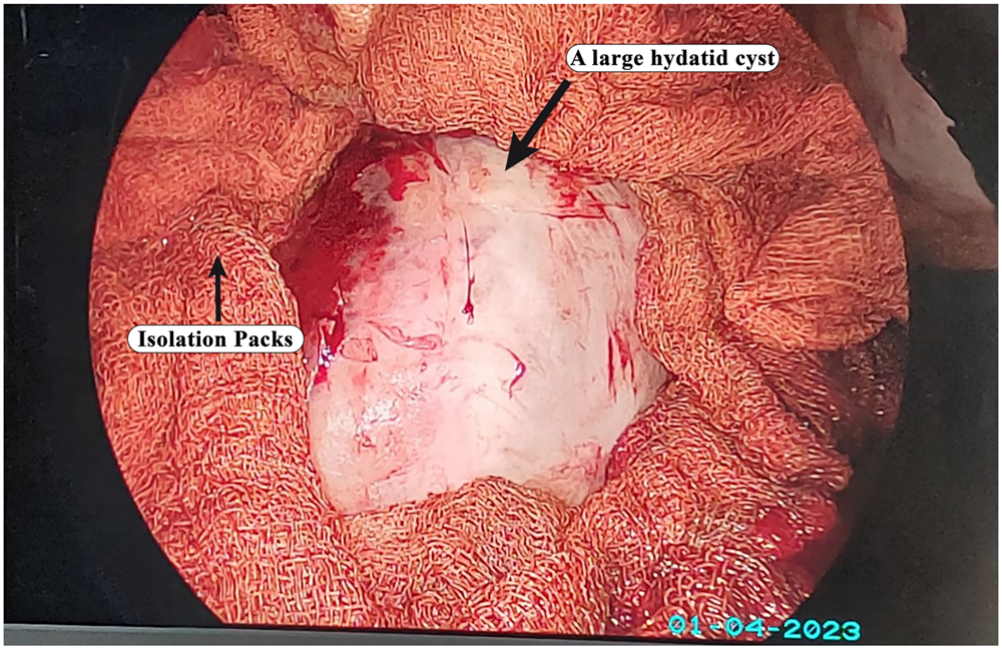
This intraoperative VATS photo shows a large hydatid cyst measuring 12 centimeters in diameter surrounded by isolation packs saturated with diluted povidone‐iodine and hypertonic saline solutions.

Before the intervention, patients received an injection of antibiotic (ceftriaxone 1 g, IV) 2 hours beforehand. The procedures were conducted under general anesthesia, with patients positioned laterally, utilizing lung exclusion and a double‐lumen endotracheal tube. In the thoracotomy group (control group), a traditional thoracotomy was done via the fifth intercostal space (20 cm), which was centered at the posterior axillary line. For the VATS group (experimental group), a 3–5 cm incision was made in the same location for both groups, the cysts were identified, isolated, and removed. The operative site was then swabbed with a hypertonic saline solution and diluted povidone‐iodine (Figure 1). Capitonnage was then performed, and hemostasis was achieved. The pleural cavity was irrigated with hypertonic saline, and a chest tube was inserted through a separate incision for both groups. At the end of the procedure, both the thoracotomy site and the utility incision were infiltrated with Marcaine (bupivacaine). The procedures were conducted by the same thoracic surgeon.

#### 2.1.6 Postoperative Care

Upon admission to the ward, intravenous antibiotics were prescribed to the patients during the hospitalization period. Pain management commenced with intravenous paracetamol (1 g × 3) and ketorolac (30 g × 3). If pain persisted, pethidine was administered subcutaneously at a dose of 50 mg. The criteria for removing the chest tube included the absence of any air leak and the presence of an expanded lung, as confirmed by physical examination and chest X‐ray. Thereafter, patients were discharged with a 5‐day course of oral antibiotics (ciprofloxacin 500 mg twice daily) and analgesics. All patients in both groups were prescribed albendazole at a dosage of 12 mg/kg, which commenced immediately after the operation and continued for 6 months. The medication was administered monthly, with a 1‐week discontinuation (3 weeks per month). Patients underwent a chest CT scan every 6 months for a duration of 2 years. The follow‐up period varied from 6 months to 3 years, with a median duration of 2 years. The “Numeric Rating Scale (NRS)” was utilized to assess pain intensity, with scores ranging from 0 to 10. A score of 0 indicated no pain, scores of 1–3 denoted mild pain, scores of 4–6 represented moderate pain, and scores of 7–10 corresponded to severe pain or the worst pain imaginable [16]. During hospitalization, the patient's pain was assessed daily. Following discharge, the patient was evaluated 12 days after the operation. Return‐to‐work data were collected during follow‐up by asking patients when they resumed work without inquiring about the nature of their jobs.

### 2.2 Statistical Analysis

The data were gathered using Microsoft Excel Sheet (2019) and analyzed by the Statistical Package for Social Sciences (SPSS) version 25. Descriptive analysis was performed, and the data were presented in terms of frequency, percentage, median, and interquartile ranges. Correlation analysis was conducted to examine the relationships between pain scores, time to work return, hospital stays, and intraoperative blood loss in relation to various demographic factors, surgical techniques, and cyst characteristics. Pearson's correlation coefficient was employed to evaluate the strength and direction of linear associations among continuous variables, while Spearman's rank correlation was utilized for data that were non‐normally distributed or ordinal. The correlation coefficients and their associated p‐values were presented to assess the statistical significance of the identified relationships, with a *p*‐value below 0.05 considered indicative of significance. Furthermore, confidence intervals for the correlation coefficients, expressed as lower and upper limits, were calculated to estimate the findings' precision and reliability.

## 3 Results

**Figure 2:**
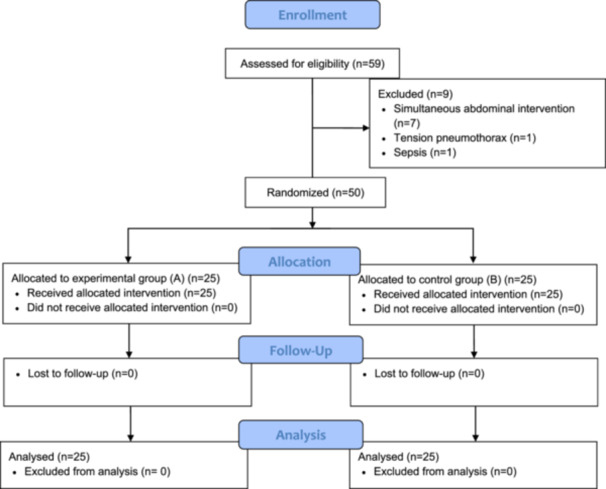
Flow chart of the enrolment of patients.

In total, 59 patients were evaluated for eligibility criteria. Seven patients were excluded from the study due to simultaneous operations for liver HC, one due to presentation with tension pneumothorax, and one due to presentation with sepsis necessitating emergency evacuation (Figure 2). A total of 50 patients diagnosed with PHC were included and equally divided into two treatment groups. Both groups exhibited a similar median age (45.5 years in thoracotomy and 43.5 years in VATS), and sex distribution (21 males and 29 females). Comorbidities such as diabetes mellitus (24% in thoracotomy and 20% in VATS) and hypertension (16% in thoracotomy and 12% in VATS) were observed in both groups. The majority of patients presented with a single cyst (60%), with a median cyst size of approximately 6.5 in the control group and 7 cm in the experimental group. Both groups demonstrated comparability in terms of primary outcomes, as two patients (8%) in the thoracotomy group and three patients (12%) in the VATS group developed intraoperative rupture (*p*‐value 0.07), and neither recurrence nor mortality was reported. Intraoperative blood loss and hospitalization period were comparable between the groups, with median values of 140 mL in the VATS group and 150 mL in the thoracotomy group for blood loss and 3 days for hospital stays. The VATS group exhibited a lesser return time to work, with a median of 18 days, and lower postoperative pain scores compared to the counterpart group (Table 1).

**Table 1:** The baseline characteristics of cases.

	Thoracotomy (frequency/percentage)	VATS (frequency/percentage)
Sample size	25	25
Median age in years (IQR)	45.50 (19–68)	43.50 (18–67)
Sex		
Male	11 (44.0%)	10 (40.0%)
Female	14 (54.0%)	15 (60.0%)
Smokers	11 (44.0%)	10 (40.0%)
Comorbidities		
DM	6 (24.0%)	5 (20.0%)
HTN	4 (16.0%)	3 (12.0%)
COPD	2 (8.0%)	1 (4.0%)
Number of cysts		
1	16 (64.0%)	14 (56.0%)
2	4(16.0%)	5 (20.0%)
≥ 3	5 (20.0%)	6 (24.0%)
Median cyst size in cm (IQR)	6.50 (3–13)	7.0 (3–12)
Median blood loss in mL (IQR)	150 (70–900)	140 (50–800)
Median hospitalization in days (IQR)	3.0 (3–6)	3.0 (3–7)
Median time to work return in days (IQR)	27.0 (19–34)	18.0 (18–23)
Pain scores		
1 (Mild)	1 (4.0%)	5 (20.0%)
2 (Moderate)	12 (48.0%)	15 (60.0%)
3 (Severe)	12 (48.0%)	5 (20.0%)

Abbreviations: COPD, chronic obstructive pulmonary disease; DM, diabetes mellitus; HTN, hypertension, IQR, interquartile range.

Correlation analysis demonstrated that VATS negatively correlated with postoperative pain score (correlation coefficient: −0.46, *p*‐value: 0.001), indicating that the experimental group was linked with lower postoperative pain. In addition, patients who underwent VATS showed a negative correlation with the return time to work (correlation coefficient: −3.86, *p*‐value: 0.001), indicating that those patients could return to work sooner than those managed by thoracotomy. A positive correlation was observed between age and blood loss, suggesting that blood loss tended to increase with advancing age (correlation coefficient: 4.88, *p* = 0.03). Other factors such as sex, comorbidities, and cyst characteristics showed no significant correlations with outcomes in either group, indicating that these factors likely did not have a considerable impact on postoperative pain, return time to work, and other surgical outcomes (Table 2).

**Table 2:** Correlation analysis of pain scores, time to work return, hospital stays, and intraoperative blood loss with operations, demographic data, and cyst characteristics.

	Pain score				Time to work return			
	Correlation coefficient	*p* value	LL	UL	Correlation coefficient	*p* value	LL	UL
VATS	−0.46	0.001^b^	−0.72	−0.20	−3.86	0.001^a^	−6.11	−1.61
Age	0.001	0.75^b^	−0.01	0.01	−0.004	0.92^a^	−0.09	0.08
Female	−0.08	0.68^b^	−0.47	0.31	−1.70	0.32^a^	−5.10	1.70
Nonsmoking	0.14	0.53^b^	−0.30	0.58	3.77	0.05^a^	−0.05	7.60
No hypertension	0.06	0.80^b^	−0.45	0.58	1.07	0.64^a^	−3.43	5.57
No diabetes mellitus	−0.14	0.48^b^	−0.55	0.26	−0.59	0.74^a^	−4.08	2.89
Cyst no.								
2	0.09	0.62^b^	−0.27	0.44	−1.29	0.41^b^	−4.37	1.79
3	−0.18	0.38^b^	−0.59	0.23	−1.29	0.47^b^	−4.83	2.25
4	−1.05	0.001^b^	−1.63	‐0.46	−5.67	0.03^b^	−10.72	−0.62
5	0.29	0.66^b^	−0.99	1.57	−14.66	0.01^b^	−25.75	−3.56
6	−0.22	0.74^b^	−1.51	1.07	3.20	0.57^b^	−8.00	14.36
7	0.31	0.62^b^	−0.95	1.58	−1.73	0.75^b^	−12.70	9.24
Cyst size								
2	0.26	0.65^b^	−0.88	1.41	6.10	0.22^b^	−3.81	16.01
3	0.07	0.88^b^	−0.82	0.96	1.03	0.79^b^	−6.70	8.76
4	−0.29	0.51^b^	−1.18	0.60	5.68	0.15^b^	−2.03	13.39
5	−0.18	0.65^b^	−0.99	0.62	0.002	1.00^b^	−6.95	6.95
6	−0.21	0.60^b^	−1.02	0.59	2.55	0.47^b^	−4.43	9.53
7	0.03	0.94^b^	−0.79	0.85	1.24	0.73^b^	−5.85	8.33
8	0.15	0.71^b^	−0.67	0.98	2.57	0.48^b^	−4.59	9.73
9	0.15	0.72^b^	−0.67	0.96	1.52	0.67^b^	−5.54	8.58
10	−0.45	0.38^b^	−1.47	0.56	3.91	0.38^b^	−4.91	12.74
11	−0.37	0.53^b^	−1.54	0.80	6.72	0.19^b^	−3.39	16.84
12	−1.10	0.20^b^	−2.66	0.46	−1.22	0.86^b^	−14.74	12.31
13	−0.70	0.36^b^	−2.22	0.82	−5.67	0.39^b^	−18.83	7.49
	**Hospital stays**				**Intraoperative blood Loss**			
VATS	0.16	0.65^a^	−0.54	0.87	4.72	0.94^a^	−128.97	119.53
Age	−0.01	0.52^a^	−0.03	0.02	4.88	0.03^a^	0.41	9.36
Female	0.14	0.79^a^	−0.92	1.21	−9.23	0.92^a^	−196.83	178.36
Nonsmoking	0.04	0.95^a^	−1.16	1.23	55.58	0.60^a^	−266.74	155.57
No hypertension	−0.31	0.67^a^	−1.71	1.10	59.27	0.64^a^	−189.16	307.70
No diabetes mellitus	0.32	0.56^a^	−0.77	1.41	124.60	0.20^a^	−67.96	317.15
Cyst no.								
2	−0.09	0.86^b^	−1.05	0.88	4.47	0.96^b^	−165.42	174.35
3	−0.07	0.90^b^	−1.18	1.04	43.02	0.66^b^	−152.58	238.63
4	−0.79	0.32^b^	−2.37	0.79	77.77	0.58^b^	−356.82	201.29
5	−1.48	0.40^b^	−4.96	1.99	220.47	0.48^b^	−833.22	392.27
6	−0.72	0.68^b^	−4.22	2.78	332.39	0.29^b^	−949.73	284.95
7	−1.63	0.35^b^	−5.06	1.81	98.45	0.75^b^	−704.32	507.41
Cyst size								
2	0.24	0.88^b^	−2.87	3.34	−9.09	0.97^b^	−556.49	538.31
3	0.93	0.45^b^	−1.49	3.35	13.90	0.95^b^	−440.65	412.84
4	0.95	0.44^b^	−1.46	3.36	25.08	0.91^b^	−400.67	450.82
5	0.89	0.42^b^	−1.28	3.07	21.25	0.91^b^	−362.54	405.03
6	0.75	0.50^b^	−1.44	2.93	153.89	0.43^b^	−231.55	539.34
7	1.46	0.19^b^	−0.76	3.68	36.25	0.85^b^	−355.33	427.82
8	1.96	0.09^b^	−0.28	4.20	12.59	0.95^b^	−407.95	382.78
9	0.89	0.43^b^	−1.32	3.10	237.07	0.23^b^	−152.80	626.92
10	2.02	0.15^b^	−0.75	4.78	173.55	0.48^b^	−313.94	661.03
11	0.12	0.94^b^	−3.05	3.29	147.84	0.60^b^	−410.75	706.43
12	−0.15	0.94^b^	−4.38	4.08	168.90	0.65^b^	−915.72	577.92
13	0.34	0.87^b^	−3.79	4.46	40.98	0.91^b^	−686.0	767.95

Abbreviations: LL, lower limit; UL, upper limit.

^a^ Pearson's correlation coefficient.

^b^ Spearman's rank correlation.

## 4 Discussion

The disease is especially prevalent among individuals engaged in animal husbandry, as frequent contact with infected dogs and livestock significantly enhances the risk of transmission. In endemic areas, the incidence may surpass 50 cases per 100,000 people yearly [17]. Countries, such as Turkey, Iran, and Greece, report high incidences of hydatidosis, with prevalence rates ranging from 3 to 10 cases per 100,000 population annually. In Argentina and Chile, incidence rates can reach up to 20 cases per 100,000 population [4, 18, 19, 20]. Patients with PHC are most commonly aged between 20 and 40 years, although the condition can affect individuals of all age groups. Men are often disproportionately affected, likely due to occupational exposure. Alternatively, it has been proposed that women may have an increased risk of infection owing to their domestic responsibilities, particularly those involving the care of livestock [21].

Due to the lack of consensus on the disease's treatment approach, this study compared the treatment outcomes between VATS and open thoracotomy in managing PHC, adding substantial data on this controversial topic. In this study, the median length of hospitalization was comparable for both groups, with a duration of 3 days. This finding was consistent with Alpay et al. who reported a mean hospital stay of 4.65 days in thoracotomy and 4 days in VATS [22]. However, this contrasts with some previous studies, which have shown that VATS significantly reduces hospitalization duration compared to thoracotomy [23, 24]. A pediatric study reported that patients undergoing VATS experienced a mean hospital stay of 10.5 days, significantly shorter than the 17.3 days observed in those who underwent thoracotomy [25]. This pattern aligns with the fact that VATS not only reduces hospital stays but also lowers overall hospitalization costs [25]. This may be due to the fact that open thoracotomy utilizes a conventional method, requiring a sizable chest wall incision to provide proper access to the diseased lung [11]. The surgeon excises the cyst(s) and may undertake additional repairs of lung tissue [26]. The findings of our study may be attributed to the small sample size. In addition to that, there was no correlation between the number of cysts and hospitalization duration, which aligns with the study by Sadrizadeh and colleagues [27], which found no notable correlation between the number of cysts and the duration of hospitalization among individuals undergoing surgical intervention for PHC. Furthermore, the number of lesions did not significantly influence the incidence of postoperative complications [28].

In the present study, the pain score (negative correlation value of −0.46) and median time to work return (negative correlation value of −3.86) were significantly lower in patients undergoing VATS than in their counterparts. These results aligned with another study indicating that patients who underwent VATS experienced lower postoperative pain scores [23]. Furthermore, in another study, correlation analysis revealed a moderate negative association between VATS and postoperative pain scores, reinforcing the evidence that VATS is linked to reduced pain levels [29]. The recovery period for returning to work has been reported to be shorter for patients following VATS compared to thoracotomy. This is attributed mainly to the minimally invasive nature of VATS. Overall, the evidence indicates that patients undergoing VATS can resume normal activities more quickly than those who undergo thoracotomy [29]. Additionally, it may yield more favorable cosmetic results, resulting in less conspicuous scarring [22, 29].

In this study, the median intraoperative blood loss was 140 mL in the VATS group and 150 mL in the thoracotomy group, a difference that was not statistically significant. In our cases, the observed VATS blood loss (50–800 mL) was lower than the values reported in the literature, where blood loss associated with VATS for PHC removal typically ranges from 400 to 700 mL [30]. Moreover, our findings contradict the assumption that VATS significantly reduces perioperative bleeding compared to thoracotomy [30]. A study by Ma et al. documented a mean intraoperative blood loss of approximately 18.30 mL for thoracotomy patients, whereas those undergoing VATS experienced significantly less, averaging 8.80 mL [23]. The comparable blood loss between the two approaches in our study may be due to the fact that the quantity of intraoperative blood loss may vary based on factors like the location of the cyst, as well as the unique characteristics of each patient [11, 30].

Factors contributing to recurrence include incomplete cyst removal and intraoperative spillage [31]. Consistent with the literature [32], none of the cases in either group experienced recurrence in the current study, as reported recurrence rates ranged from 0% to 2.7% among patients undergoing thoracotomy. Another study investigating the long‐term outcomes of VATS reported that no recurrences were observed among patients treated for bilateral hydatid disease, even during follow‐up periods extending up to 23 years [33]. However, the lack of recurrence in the present study may be influenced by the relatively short follow‐up period of 2 years. The findings of this study may have significant clinical implications, particularly in optimizing surgical decision‐making for PHC. The comparable hospitalization duration between VATS and thoracotomy suggests that while VATS offers a minimally invasive approach, its impact on hospital stay may be influenced by patient‐specific factors. However, the significantly lower postoperative pain scores and shorter return‐to‐work times highlight its advantages in enhancing recovery, reducing opioid dependence, and improving quality of life. Additionally, the comparable intraoperative blood loss challenges the assumption that VATS universally reduces perioperative bleeding, emphasizing the need for patient selection based on cyst characteristics and surgical expertise. Future research should focus on multicenter studies with larger cohorts to improve generalizability, long‐term follow‐up to assess recurrence rates, and cost‐effectiveness analyses to determine the economic viability of VATS. Further investigations into optimized pain management strategies, predictive models for patient selection, and innovations such as robotic‐assisted techniques could refine treatment approaches and enhance patient outcomes.

Several limitations warrant acknowledgment. Firstly, the sample size might not sufficiently encapsulate the entirety of individuals undergoing PHC surgery. Utilizing a larger sample size would yield more accurate and broadly applicable results. Secondly, the study was based on a single center, potentially constraining the extrapolation of conclusions to other facilities with dissimilar patient demographics and surgical methodologies. Additionally, there was a lack of an objective comparison of the clinical and radiological characteristics between the two groups, particularly in terms of cyst sizes and features. Finally, the follow‐up period might not be enough to evaluate the recurrence rate accurately.

## 5 Conclusion

VATS shows promise in reducing postoperative pain and facilitating a sooner work resume than open thoracotomy. However, surgical outcomes in both groups may not be affected by variables such as age (except for blood loss), sex, or concurrent health conditions.

## Author Contributions

**Fahmi H. Kakamad:** conceptualization, data curation, formal analysis, investigation, methodology, validation, visualization, writing – original draft, writing – review and editing.

## Conflicts of Interest

The author declares no conflicts of interest.

## Transparency Statement

The lead author Fahmi H. Kakamad affirms that this manuscript is an honest, accurate, and transparent account of the study being reported; that no important aspects of the study have been omitted; and that any discrepancies from the study as planned (and, if relevant, registered) have been explained.

## Supporting information

CONSORT 2010 Checklist.

## Data Availability

The author confirms that the data supporting the findings of this study are available within the article [and/or] its Supporting Information.
